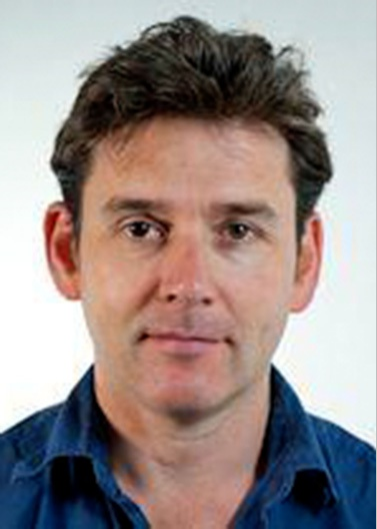# NAD(P)H‐Dependent Dehydrogenases for the Asymmetric Reductive Amination of Ketones: Structure, Mechanism, Evolution and Application

**DOI:** 10.1002/adsc.201700356

**Published:** 2017-05-11

**Authors:** Mahima Sharma, Juan Mangas‐Sanchez, Nicholas J. Turner, Gideon Grogan

**Affiliations:** ^1^ York Structural Biology Laboratory Department of Chemistry University of York YO10 5DD York U.K.; ^2^ School of Chemistry University of Manchester Manchester Institute of Biotechnology 131 Princess Street Manchester M1 7DN UK.

**Keywords:** amination, amine dehydrogenase, enzyme catalysis, imine reductase, oxidoreductases, reductive amination

## Abstract

Asymmetric reductive aminations are some of the most important reactions in the preparation of active pharmaceuticals, as chiral amines feature in many of the world's most important drugs. Although many enzymes have been applied to the synthesis of chiral amines, the development of reductive amination reactions that use enzymes is attractive, as it would permit the one‐step transformation of readily available prochiral ketones into chiral amines of high optical purity. However, as most natural “reductive aminase” activities operate on keto acids, and many are able to use only ammonia as the amine donor, there is considerable scope for the engineering of natural enzymes for the reductive amination of ketones, and also for the preparation of secondary amines using alkylamines as donors. This review summarises research into the development of NAD(P)H‐dependent dehydrogenases for the reductive amination of ketones, including amino acid dehydrogenases (AADHs), natural amine dehydrogenases (AmDHs), opine dehydrogenases (OpDHs) and imine reductases (IREDs). In each case knowledge of the structure and mechanism of the enzyme class is addressed, with a further description of the engineering of those enzymes for the reductive amination of ketones towards primary and also secondary amine products.

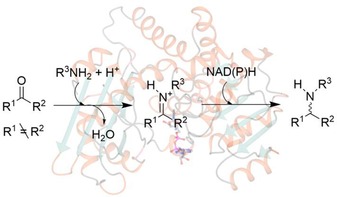

## 1 Introduction

**Scheme 1:**
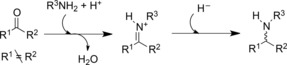
General scheme illustrating the reductive amination reaction.

Reductive amination, or the conversion of a carbonyl group to an amine *via* an iminium ion intermediate (Scheme 1), is one of the most important reactions for synthesising chiral amines, a functional group that features in a considerable proportion of small biologically active molecules.

Moreover, an increasing amount of research is being directed towards the development of asymmetric processes for reductive amination,^1^,^2^ as, in many cases, the stereocentre bearing the amine is crucial in determining its biological activity. Examples of abiotic asymmetric reductive amination include organometallic catalysis using different transition metal complexes, such as ruthenium, rhodium and iridium, employing either H_2_ 3,4 or other reducing agents.^5^ These techniques have been extensively applied and are well‐established in industry. However, they also present serious environmental and safety issues due to the use of transition metals and hydrogen gas, frequently under high pressures. Organocatalytic approaches^6^ have also been employed using either hydrosilanes^7^ or Hantzsch esters^8^ as the hydrogen source, utilising chiral Brønsted acid species such as Akiyama–Terada catalysts to induce chirality.

**Figure 1:**
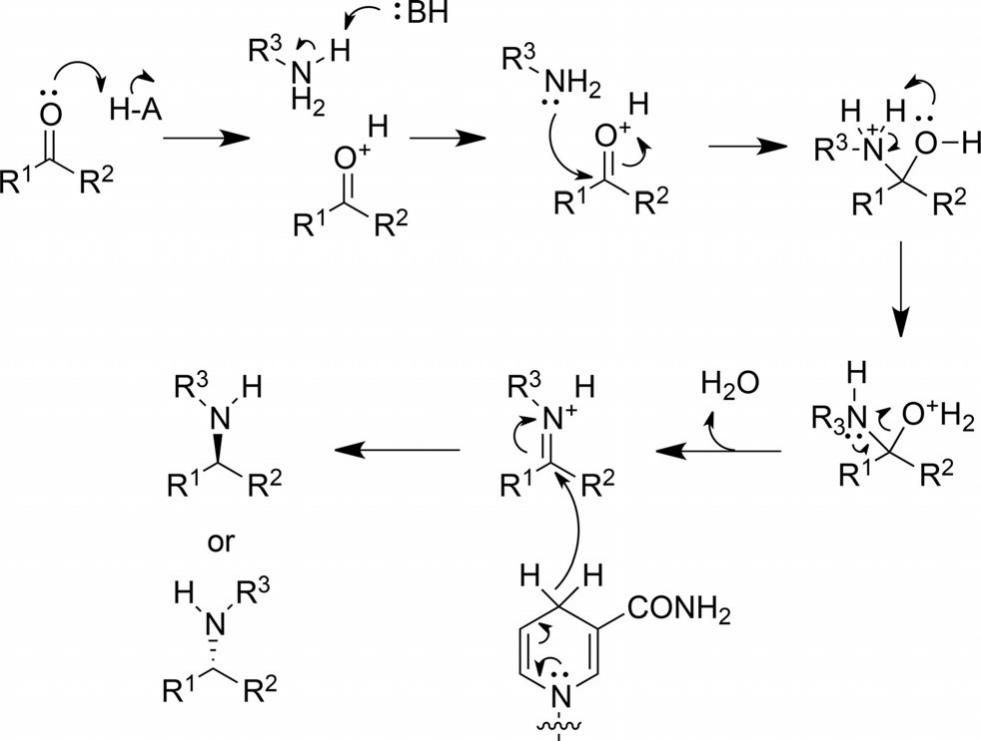
Putative mechanism for an enzyme‐catalysed reductive amination of a ketone.

When the optical purity of products is of paramount importance, then biocatalytic routes to these compounds also command consideration.^9^ The list of enzymes that are used to catalyse the synthesis of chiral amines now stretches from hydrolases^10^ for the resolution of *N*‐acylamines, through to flavin‐dependent monoamine oxidases (MAOs) for the deracemisation of chiral amines,^11^ and ω‐transaminases (ω‐TAs),^12^ which are able to synthesise chiral amines from ketones at the expense of an ammonia donor, such as alanine or isopropylamine. The last example is interesting from the current perspective, in that ω‐TAs catalyse formal reductive amination reactions, although no reductive chemistry is involved. However, despite many important examples of the application of ω‐TAs in amine production, the enzymes have limitations that are dictated by their mechanism. In a transamination, ammonia from the donor is transferred to the enzyme cofactor pyridoxal 5‐phosphate (PLP), which retains the ammonia within the modified cofactor pyridoxamine 5‐phosphate (PMP). The ammonia is transferred to the co‐substrate of the reaction, in this case the prochiral ketone that will be converted into an amine. Only transfer of ammonia is permitted by this system, and, hence, the syntheses using transaminases are limited to the synthesis of primary amines. The enzymatic preparation of chiral secondary amines has been achieved using MAOs,^11^ although a racemic amine itself is the starting material. Recent advances in imine reductase (IRED) biocatalysis,^13^,^14^,^15^ addressed below, also permit the preparation of chiral secondary amines from preformed prochiral imines. However, in those cases, the necessary preparation of imine substrates itself presents a constraint. A preferred reaction would be the enzymatic reductive amination of a prochiral ketone, in which an enzyme would catalyse bond formation between the ketone and amine, and subsequent reduction of the iminium ion intermediate. The mechanism of a true ‘reductive aminase’ enzyme would mirror that of the abiotic equivalent in which an enzyme would catalyse the coupling of ketone and amine substrates, presented in a 1:1 ratio, to form a carbinolamine intermediate, from which water was eliminated to form a iminium ion (Figure 1). The enzyme would then be able to asymmetrically reduce the prochiral iminium ion using a nicotinamide cofactor [NAD(P)H] to generate a chiral amine product. Such an enzyme would also require a mechanism to prevent reduction of the keto precursor to the alcohol. ‘Reductive Aminases’ (RedAms) competent for the true reductive amination of ketones, would therefore be extremely useful additions to the selection of biocatalytic agents for the production of chiral amines.

In this review we describe the inspiration for the design of such catalysts based on natural reductive amination enzymes that act on keto acid substrates. We also discuss examples in which native activities can be employed in reductive amination processes, and discuss how these might be evolved for improved RedAm activity. In each case the structure, mechanism, and the application of the enzyme to amine synthesis, are considered in the description.

## 2 Amino Acid Dehydrogenases and their Evolution for the Reductive Amination of Ketones

**Scheme 2:**
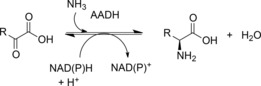
General reaction catalysed by amino acid dehydrogenases (AADHs).

Amino acid dehydrogenases (AADHs, E.C. 1.4.1.X) catalyse the NAD(P)H‐dependent interconversion of keto and amino acids (Scheme 2).^16^ In the amination direction, the amino acid is formed through the enzyme‐catalysed coupling of the relevant keto acid and ammonia, followed by reduction of the imino acid intermediate using hydride supplied by the cofactor. A vast history exists in the literature on the biochemistry, but also on the applications of AADHs,^17^,^18^ and many mechanistic studies on the chemistry through which the reductive amination is achieved, have been published.

The most extensively studied AADHs deaminate leucine (LeuDH),^19^ phenylalanine (PheDH),^20^ glutamic acid (GluDH)^21^ and also *meso*‐diaminopimelate (*meso*‐DAPDH),^22^ although additional enzymes active in the conversion of glycine, alanine, aspartic acid, lysine and tryptophan have also been reported.^18^ The potential for engineering these enzymes for the reductive amination of ketones has been best realised for the examples of LeuDH and PheDH.

### 2.1 Structure and Mechanism of LeuDH

**Scheme 3:**
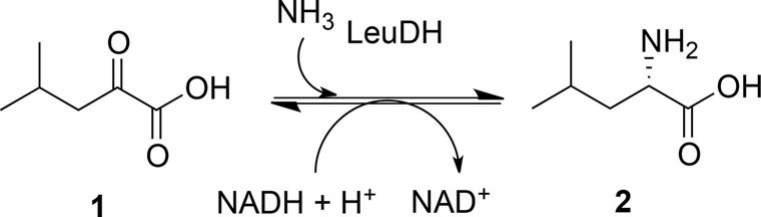
Activity of wild‐type LeuDH.

LeuDH (E.C.1.4.1.9) catalyses the reversible reductive amination of 4‐methyl‐2‐oxopentanoate (α‐keto isocaproate) **1** to leucine **2** (Scheme 3) and is NADH‐dependent.

Further activities towards α‐ketobutyrate and α‐ketovalerate have been reported in homologues from *Bacillus sphaericus* 23 and *Bacillus stearothermophilus*.^24^ Native LeuDH has an established history in industrial biotransformations for the production of *tert*‐leucine, an essential component of viral protease inhibitors.^25^,^26^

**Figure 2:**
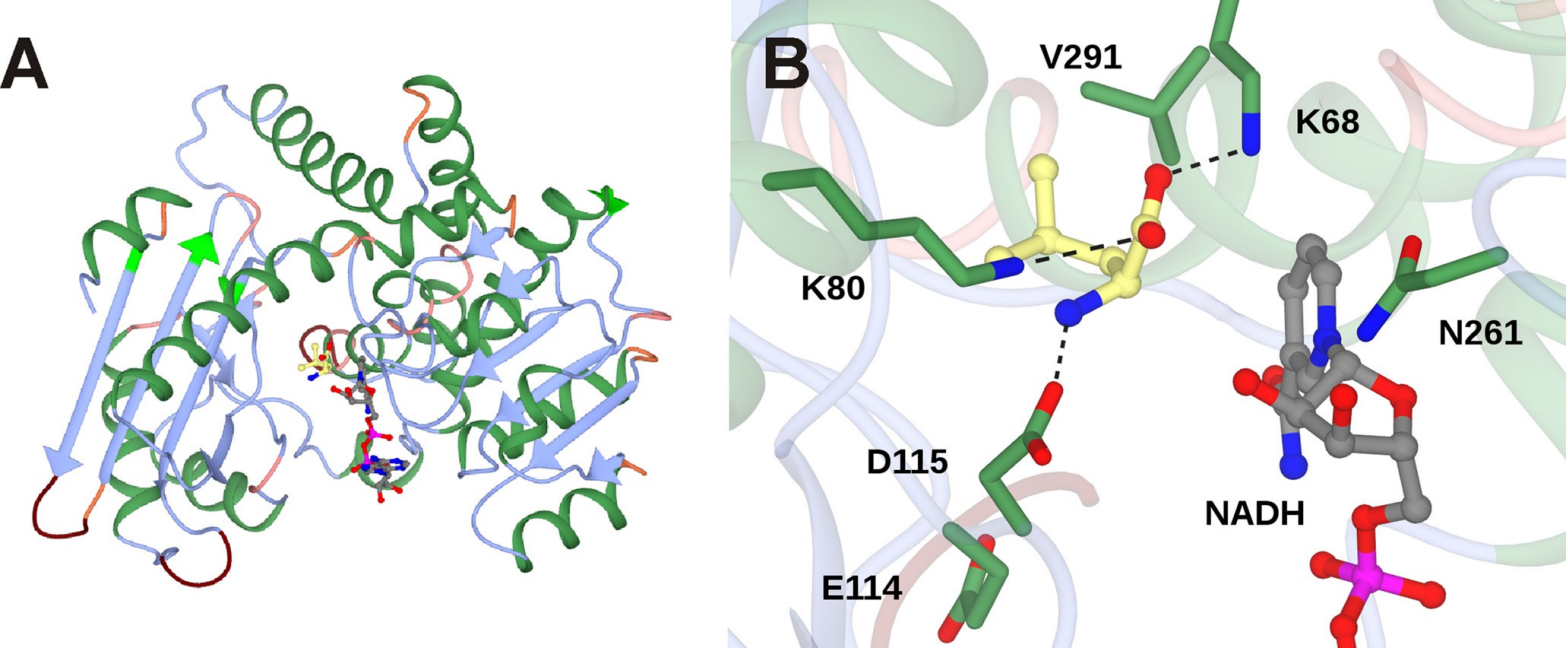
**A**: Structure of monomer of LeuDH (1LEH),^27^ with NADH (carbon atoms in grey) and l‐leucine (carbon atoms in yellow) modelled within the active site. **B**: Detail of active site of LeuDH showing significant interactions between active site side‐chains and l‐leucine, and residues targeted for mutation in directed evolution studies.

The structure of the LeuDH homologue from *B. sphaericus* was determined by Rice and co‐workers (PDB 1LEH).^27^ One subunit of the enzyme is 364 amino acids and is organised into two domains separated by a deep cleft in which the cofactor binds (Figure 2A). In the crystal structure two subunits associate to form a dimer, but the quaternary structure of LeuDH is thought to be an octamer in solution. It was thought that domain movements play a role in catalysis, with a closure of the domains in the presence of the substrate required to bring the cofactor and substrates sufficiently close for hydride transfer.

Although the authors report a complex with the cofactor NAD(H), the atom coordinates for this molecule are not contained within the relevant PDB file. However, the report details the determinants of cofactor binding within the binding cleft, and a comparison with the structures of phenylalanine dehydrogenase (*vide infra*) bound to both NAD(H) and phenylalanine permits a model to be constructed that places both NAD(H) and l‐leucine within the active site (Figure 2B).

**Figure 3:**
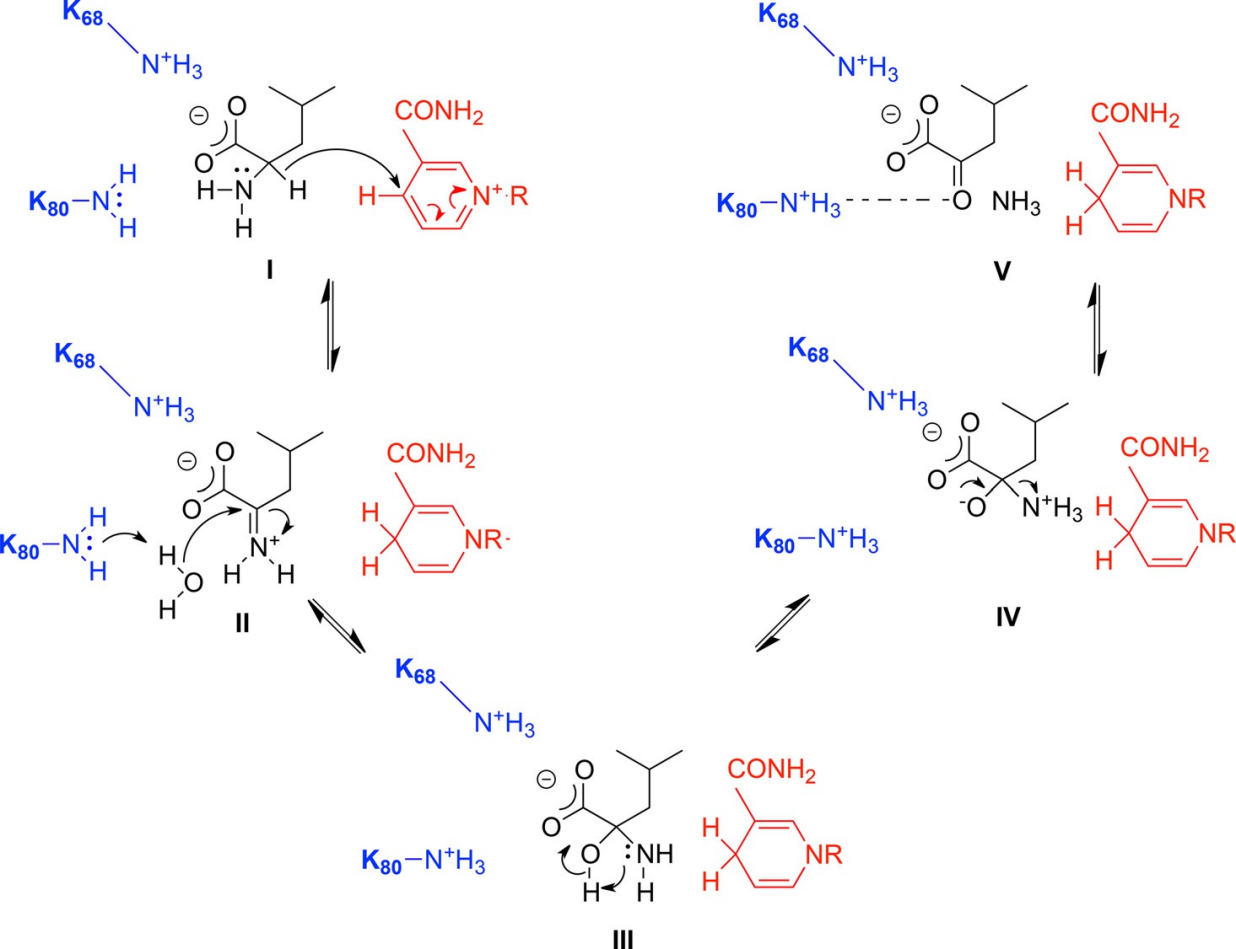
Proposed mechanism of deamination of l‐leucine by LeuDH adapted from Sekimoto and co‐workers.^28^ **I**: l‐leucine; **II**: iminium ion; **III**: carbinolamine; **IV**: oxyanion; **V**: α‐ketoisocaproate.

The model suggests that l‐leucine is secured in the active site through the interaction of its amino group with aspartic acid residue D115 and its carboxylate with the side‐chains of two lysine residues K68 and K80 (Figure 2B). K80 is highly conserved amongst AADH sequences. An increase in *K*_m_ for α‐keto isocaproate, from 0.9 mM for the wild‐type, to 9.8 mM and 25 mM for mutants K80A and K80Q, respectively, determined by Sekimoto and co‐workers,^28^ was thought to be consistent with a role for K80 in keto acid substrate binding. The *K*_m_ for l‐leucine again increased from 5.1 mM for the wild‐type to 17 mM for K80Q, but decreased slightly to 3.7 mM for K80A. Sekimoto and co‐workers also determined that K80 acts as a general base in catalysis, most likely involved in the activation of water for attack at the electrophilic carbon of the imininum ion in the deamination direction. The model may be considered in conjunction with a proposal for the oxidative deamination of leucine put forward by Sekimoto and co‐workers shown in Figure 3.^28^ Hydride abstraction from leucine (**I)** by NAD^+^ first creates an iminium ion (**II**). K80 acts as a base, activating a water molecule for attack at the electrophilic carbon atom, to form the carbinolamine intermediate (**III**). Intramolecular proton transfer from the intermediate hydroxyl to the amine forms an oxyanion (**IV**), stabilised by the side chain of K80, and the ammonia leaving group, which departs to leave the keto acid product α‐ketoisocaproate (**V**).

### 2.2 Structure‐Guided Evolution of LeuDH for the Reductive Amination of Ketones

**Scheme 4:**
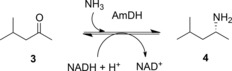
Activity of AmDH evolved from LeuDH.^29^.

The activity of LeuDH was to provide the major inspiration for the engineering, by directed evolution of an “amine dehydrogenase” (AmDH) enzyme that would be capable of reductive amination of the ketone analogue of α‐ketoisocaproate **1**, methyl isobutyl ketone **3** (MIBK, Scheme 4).^29^

Bommarius and co‐workers targeted the determinant residues of carboxylate recognition in a LeuDH from *Bacillus stearothermophilus* for mutation. Although K80 had to be retained, as it was essential for the mechanism, a saturation mutagenesis library of K68 yielded a variant, K68M, that possessed low but measurable reductive amination activity towards MIBK.^29^ Other residues, homologous with those of LeuDH from *B. sphaericus* illustrated in Figure 2B, were targeted in a mutagenesis strategy in which libraries of variants were screened for improved AmDH activity towards **3**. The most active variants resulting from the screen included those with mutations to positions K68, D114 and V291, but also N261, which is involved in the recognition of the cofactor NADH. The superior variant overall was K68S/E114V/N261L/V291C, which displayed a *k*_cat_ value of 0.46 s^−1^ for the transformation of **3** to (*R*)‐1,3‐dimethylbutylamine (1,3‐DMBA) **4** with 92.5% conversion and 99.8% *ee*, and with negligible residual activity towards l‐leucine. Activity was also recorded towards other ketones, including cyclohexanone and acetophenone.

### 2.3 Structure and Mechanism of PheDH

Further developments in this area were achieved through evolution of another AADH, phenyalanine dehydrogenase (PheDH, E.C.1.4.1.20) from *Bacillus badius*,^30^ which catalyses the interconversion of l‐phenylalanine and phenylpyruvate, again using NADH as the cofactor. PheDH shares 48% sequence identity with LeuDH, and a structure of a bacterial enzyme from *Rhodococcus* sp. M4, upon which engineering has been based, was determined by Holden, Thoden and co‐workers.^31^,^32^ The structure is very similar to that of LeuDH, consisting of two domains, separated by a large cleft. In contrast to LeuDH however, structures have been obtained in which both cofactor and the substrates of amination and deamination reactions, phenylpyruvate and l‐phenylalanine (1BW9, 1C1D) respectively, phenyllactate (1C1X) and an inhibitor, β‐phenylpropionate (1BXG), have been trapped in the active site. The catalytic activity of PheDH was again believed to be enabled by a relative closure of domains over the active site, in order to bring the substrate and cofactor into sufficiently close proximity for reaction.

**Figure 4:**
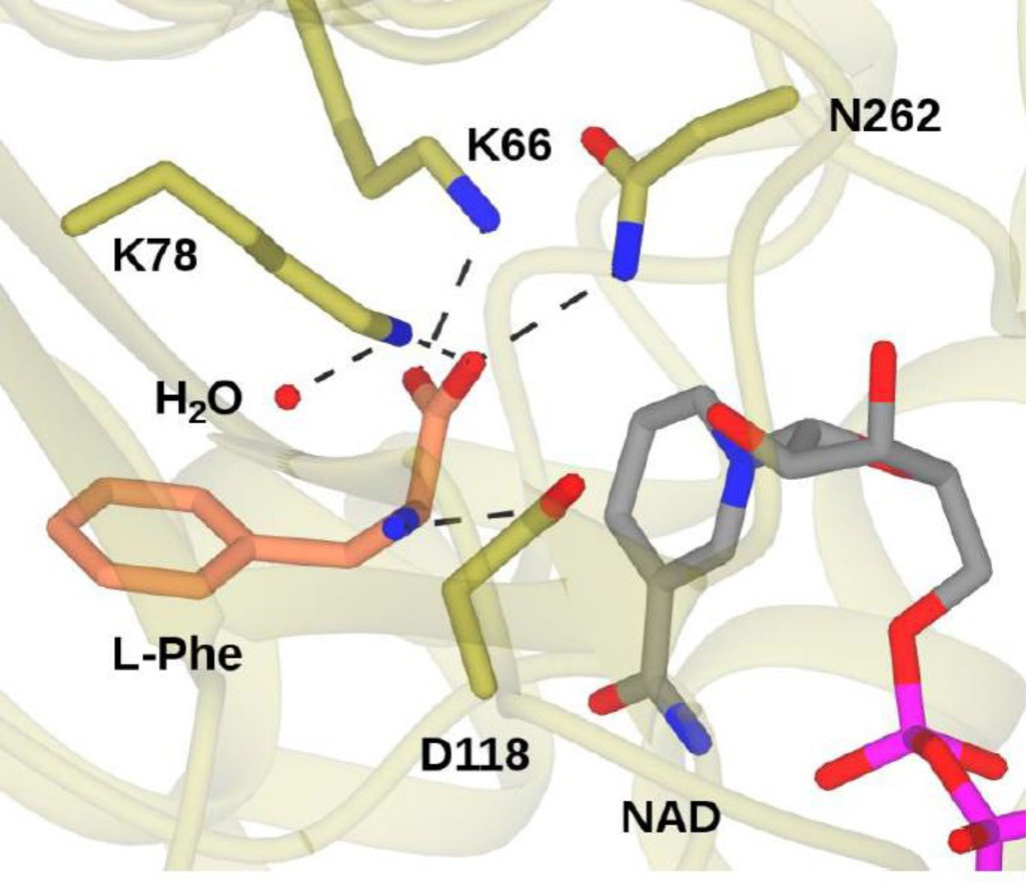
Active site of PheDH from *Rhodococcus* sp. M4 in complex with l‐Phe and NAD (PDB code 1C1D)^32^ showing interactions of the substrate with active site residues and also the catalytic water molecule.

A representation of the active site of *Rhodococcus* sp. PheDH in complex with l‐Phe is shown in Figure 4. The determinants of amino acid binding are similar to that of LeuDH, with the amino group of the substrate secured by D118 and the carboxylate making interactions with the side‐chains of two lysine residues, K66 and K78, and also N262. However, kinetic measurements using the inhibitor 3‐phenylpropionate suggest that the interaction of the amino group with D118 is not essential for substrate binding.^32^ The presence of the ligand was accompanied by the identification of a water molecule in the structure, in close proximity to the catalytic K68 side‐chain, and thought to be the one involved in formation of the carbinolamine intermediate. The mechanism in the deamination direction was defined as “ordered bi‐ter”, in which binding of the cofactor NAD^+^ was followed by that of l‐Phe, followed by ordered release of ammonium ions, phenylpyruvate and NADH.

**Figure 5:**
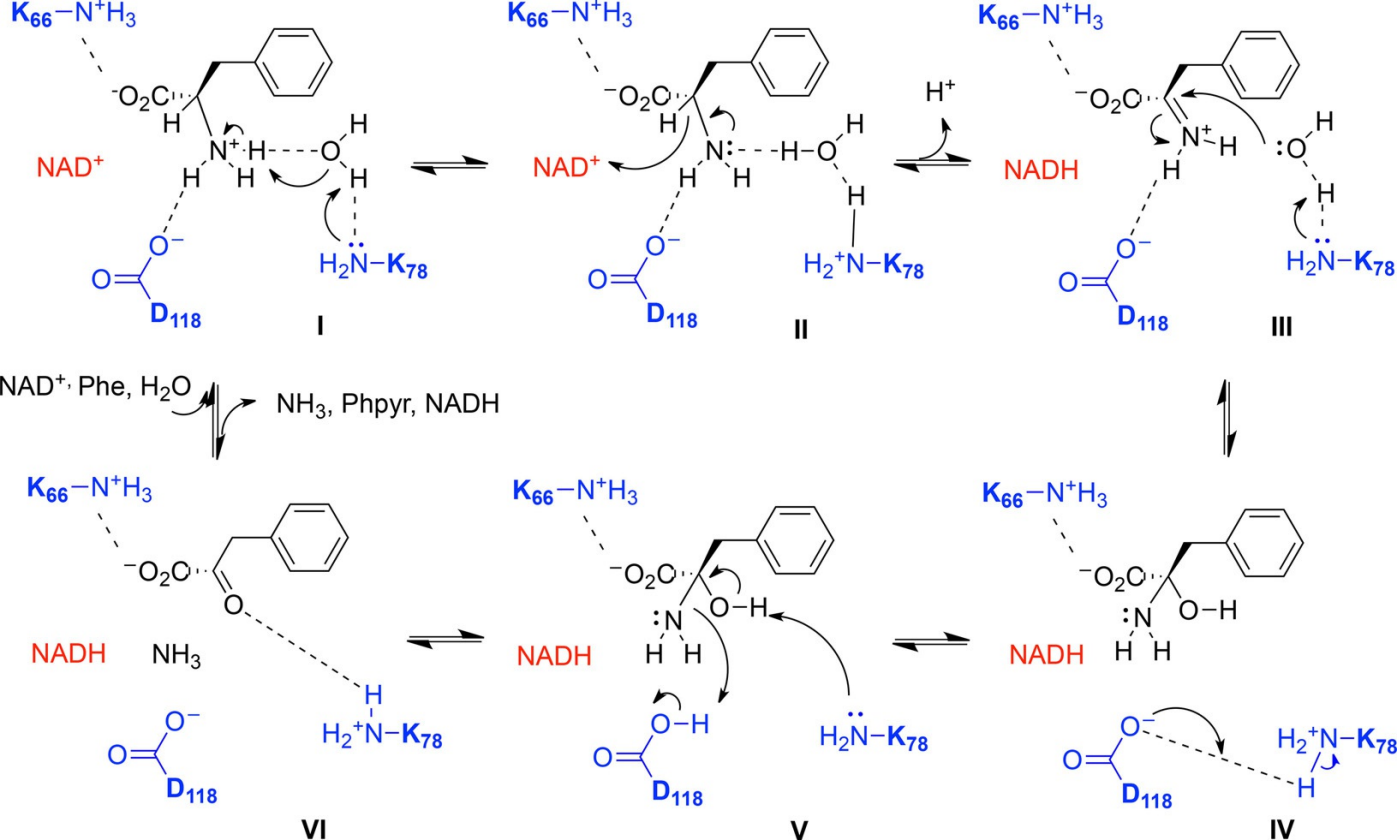
Mechanism of PheDH adapted from Brunhuber and co‐workers.^32^ Upon binding of l‐Phe (**I**), K78 catalyses deprotonation of the α‐amino group *via* a water molecule. Hydride is transferred to NAD^+^ (**II**), to form an iminium ion (**III**), which is attacked by a water molecule, activated by K78, to form the carbinolamine intermediate (**IV**). D118 protonates the departing amino group (**V**) to yield phenylpyruvate and ammonia (**VI**).

Detailed kinetic measurements led to a mechanistic proposal for the deamination, which was similar to that proposed for LeuDH (Figure 5),^32^ although with many refinements, especially with respect to proton transfer steps. In the first case, the substrate is suggested to bind in the zwitterionic form, with the amine in the protonated state. K78 activates the catalytic water molecule for deprotonation of the ammonium group (**I**), after which hydride is transferred to NAD^+^ (**II**) with formation of the iminium ion (**III**). K78 then activates water for attack at the iminium carbon (**III**), giving the carbinolamine intermediate (**IV**). K78 now acts as a base, deprotonating the carbinolamine (**V**), as D118 donates a proton to the amine leaving group. This leaves the phenylpyruvate product with its carbonyl group bound to the side chain of K78 (**VI**) Intriguingly, a comparison of the structures of PheDH in complex with phenyllactate and l‐Phe permit the authors to propose a mechanism whereby the keto acid substrate of reductive amination is prevented from being reduced to phenyllactate by the cofactor. In the phenyllactate complex, binding of the keto group of the ligand to the side‐chain of K78 positions the carbonyl carbon 5.1 Ångstrom from the C‐4 of NAD(H), is too far for hydride delivery to occur between them.

### 2.4 Evolution of PheDH for the Reductive Amination of Ketones

Bommarius and co‐workers followed the *in vitro* evolution of LeuDH with experiments designed to evolve PheDH for reductive amination of ketones.^30^ Starting with the successful LeuDH mutations as a guide, a PheDH from *Bacillus badius* was mutated to give the double variant K77M/N276V, which displayed increased activity towards MIBK over the wild‐type enzyme, but also for *p*‐fluorophenylacetone.^30^ A focused mutagenesis strategy, targeting these two positions furnished an improved variant K77S/N276L that displayed a *k*_cat_ value 15‐fold greater than that observed for the LeuDH amine dehydrogenase mutant. The new variant (F‐AmDH) displayed reductive amination activity towards a range of ketones including phenoxy‐2‐propanone, 2‐hexanone and 3‐methyl‐2‐butanone.

**Scheme 5:**
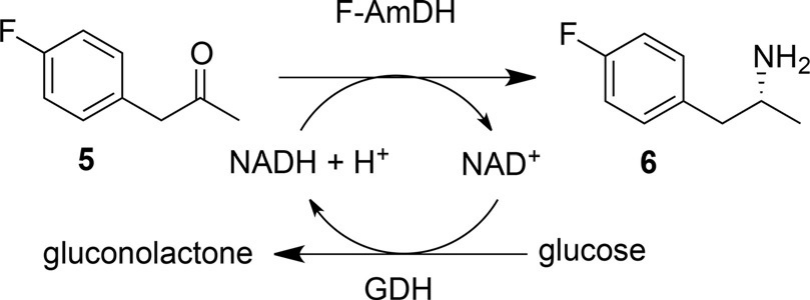
Reductive amination of *p*‐fluorophenylacetone by F‐AmDH, with cofactor recycling.^30^.

The transformation of *p*‐fluorophenylacetone **5** gave the amine product with 93.8% conversion (73.9% isolated yield) and >99.8% *ee* (Scheme 5). F‐AmDH was also compatible with the NADH recycling system of glucose and glucose dehydrogenase.

Further functionality in the new amine dehydrogenase scaffold was achieved by combining the N‐terminal section (residues 1–149) of the F‐AmDH with residues 140–166 of the LeuDH variant to give a chimeric enzyme, cFL1‐AmDH that displayed activity towards acetophenone and adamantyl methyl ketone, and converted these to (*R*)‐amine products with excellent enantioselectivity.^33^ In a further refinement, two adjacent asparagine residues in the chimera, N270 and N271, were mutated to leucine in an effort to affect amination activity. The new variant displayed increased an *k*_cat_ value towards *p*‐fluoroacetophenone.

Some of the lower activities of F‐AmDH were attributed to the poor solubility of substrates in aqueous solution. This prompted the formulation of a biphasic reaction system in which the enzyme operated in a 1:4 ratio of heptane to water.^34^ In such systems, the volumetric productivity of the F‐AmDH‐catalysed amination of *p*‐fluorophenylacetone was doubled.

**Scheme 6:**
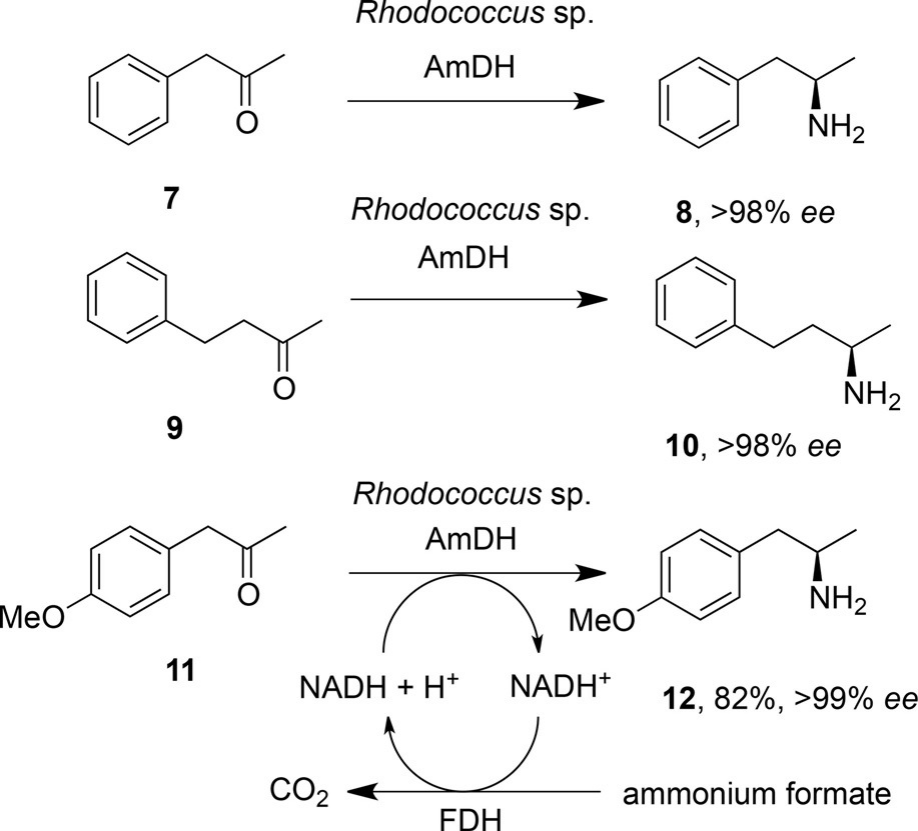
Reductive amination of ketones by AmDH created from PheDH from *Rhodococcus* sp. M4.^35^,^37^.

In subsequent work by the group of Li, the *Rhodococcus* sp. PheDH was evolved for the asymmetric reductive amination of ketones.^35^ In this report, the structure of *Rhodococcus* sp. PheDH again suggested that mutation of K66 and N262, known to interact with the natural substrate‘s carboxylate group, could be targeted in an effort to generate an enzyme that transformed ketone substrates. A focused mutagenesis strategy resulted in a triple mutant enzyme, K66Q/S149G/N262C, which catalysed the amination of phenylacetone **7** and 4‐phenyl‐2‐butanone **9** to (*R*)‐amphetamine **8** and (*R*)‐1‐methyl‐3‐phenylpropylamine **10** respectively, each with >98% *ee* (Scheme 6).

In addition to the established mutations changing the interaction with the substrate's carboxylate group, the addition of mutation S149G was thought to enlarge the size of the entrance to the substrate binding pocket. The *Rhodococcus* sp. amine dehydrogenase also proved to be compatible with the glucose/glucose dehydrogenase NADH recycling system, and 15 mM of **9** were transformed to (*R*)‐1‐methyl‐3‐phenylpropylamine **10** after 60 h, with 95.2% conversion to the product. Further work by this group saw the AmDH co‐immobilised with GDH on magnetic nanoparticles (MNPs),^36^ giving a superior system for the asymmetric reductive amination of **9** with a total turnover number for NADH recycling of 2940. A subsequent report by Mutti and co‐workers revisited the *Rhodococcus* sp. M4 amine dehydrogenase, and showed that, in addition to substrates **7** and **9**, the enzyme was also effective in transforming *o*‐methoxyphenylacetone derivatives, aliphatic ketones, such as 2‐octanone (99% conversion) and also “bulky‐bulky” ketones such as 1‐phenyl‐butan‐2‐one (>99%), 1‐phenylpentan‐2‐one (71%) and 1‐phenylpentan‐3‐one (83%).^37^ Moreover, the *Rhodococcus* sp. AmDH proved to be compatible with the more atom‐efficient formate dehydrogenase as a method for recycling the NAD^+^ cofactor. In a preparative‐scale experiment, 208 mg of (*p*‐methoxyphenyl)acetone **11** were converted to the (*R*)‐amine **12** with an 82% isolated yield and with >99% *ee* in ammonium formate buffer (Scheme 6).

### 2.5 Application of AmDHs in Cascades

**Scheme 7:**
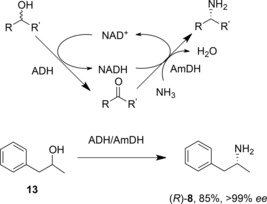
Hydrogen borrowing cascade, featuring ADH and AmDH in a closed loop system for cofactor recycling.^38^.

The F‐AmDH variant of PheDH has been applied in cascade reactions with alcohol dehydrogenases (ADHs) that permit the conversion of alcohol substrates into amine products with “closed‐loop” recycling of the cofactor (Scheme 7).^38^ In these experiments, ADHs from either *Aromatoleum aromaticum* or *Lactobacillus brevis*, which have stereocomplementary selectivities, were combined with the *Bacillus badius* PheDH variant K78S/N277L in ammonium chloride buffer at pH 8.7, with an alcohol substrate concentration of 20 mM and NAD^+^ cofactor at 1 mM. After 24 h, 85% conversion of the alcohol **13** to the (*R*)‐amine **8** was achieved, with >99% *ee*.

The technique was applicable to a wide range of 1‐phenyl‐2‐propanol derivatives. Interestingly, this “hydrogen borrowing” system permits the use of (*S*)‐, (*R*)‐ or racemic alcohol substrates, depending on which ADH, or a combination of both, is used to catalyse the alcohol oxidation.

A similar cascade was later reported by Xu and co‐workers.^39^ In this case, a LeuDH homologue from *Exiguobacterium sibiricum* (*Es*LeuDH) was engineered to contain equivalent mutations (K77S/N270L) to those engineered into the *B. stearothermophilus* LeuDH by Bommarius and co‐workers.^29^ This variant was coupled with an ADH from *Streptomyces coelicolor* that was selected on the basis of its lack of stereoselectivity for racemic substrate alcohols. A range of racemic alcohols, including 2‐pentanol and *sec*‐phenylethanol, was converted to (*R*)‐amine products with 94% and 21% conversion, respectively, and in up to >99% *ee*.

### 2.6 A Wild‐Type Amine Dehydrogenase

**Scheme 8:**
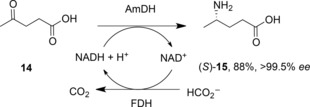
Reductive amination of 4‐oxopentanoic acid by naturally occurring amine dehydrogenase from *Petrotoga mobilis*.^40^.

The discovery, through mutation of AADHs, of AmDHs, prompted a search for natural enzymes that might possess that activity. In a search that excluded dehydrogenase homologues that act on amines with α‐ or β‐carboxylate groups, Vergne‐Vaxelaire and co‐workers used the sequence of l‐*erythro*‐3,5‐diaminohexanoate dehydrogenase (3,5‐DAHDH) as a model to identify such possible enzymes.^40^ Of 169 homologues, twenty were cloned and expressed, and of these, three were shown to aminate both 4‐oxopentanoic acid **14** (Scheme 8). and 5‐oxohexanoic acid. One homologue, AmDH4 from *Petrotoga mobilis*, was used in a semi‐preparative reaction in which 4‐oxopentanoic acid **14** was converted to (*S*)‐4‐aminopentanoic acid **15** in an isolated yield of 88% and with >99.5% *ee*, with cofactor recycling using the formate dehydrogenase system. No structure of a close homologue of this enzyme is available yet, but these naturally occurring AmDHs offer promise as additional enzyme targets for rational engineering for amine production.

## 3 Opine Dehydrogenases and their *in vitro* Evolution for the Reductive Amination of Ketones

**Scheme 9:**
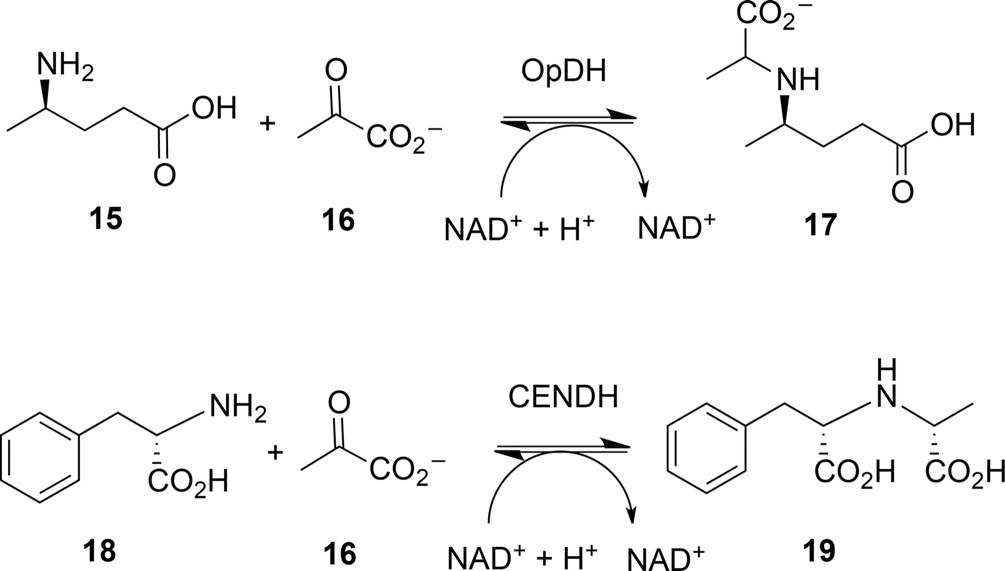
Reductive amination reactions catalysed by opine dehydrogenases OpDH such as CENDH from *Arthrobacter* sp. strain 1C.^44^.

Opine dehydrogenases (OpDHs, E.C.1.5.1.28)^41^ are a class of AADHs that catalyse the reversible reductive coupling of amino acid and keto acids to form *N*‐derivatised amino acids called opines. The model reaction catalysed by these enzymes in the BRENDA database (http://www.brenda-enzymes.org) is the NADH‐dependent coupling of aminopentanoic acid **15** and pyruvate **16** into (2*S*)‐2‐{[1‐(*R*)‐carboxyethyl]amino}pentanoate **17** (Scheme 9). They can be found in bacteria such as *Agrobacterium*,^42^ where they can be flavin dependent, and known to catalyse the formation of opines that form in crown gall tumours in infected plants, but also in higher organisms, such as molluscs and sponges, in which they are NAD(P)H‐dependent, and are thought to have a role in maintaining glycolytic flux during hypoxic conditions.^43^ They have aroused interest for applications in preparative biocatalysis, as, unusually amongst AADHs, they are able to couple a carbonyl compound with an amine that is larger than ammonia, with possible consequences for the formation of secondary amines by engineered variants.

### 3.1 Structure and Mechanism of OpDHs

The enzyme from *Arthrobacter* sp. strain 1C was discovered by Asano and co‐workers.^44^ The substrate *N*‐[1‐(*R*,*S*)‐(carboxyl)ethyl]‐*S*‐phenylalanine **19** (Scheme 9) was used as an enrichment substrate and experiments yielded the *Arthrobacter* strains from which the enzyme was successfully purified.

The enzyme – CENDH – catalysed the formation of **19** from l‐phenylalanine, sodium pyruvate and NADH as cofactor. Other hydrophobic l‐amino acids including l‐methionine, were tolerated as substrates, as well as d‐leucine, although with only 3.4% of the activity observed with l‐Met. Using l‐Met as the amino acid substrate, the enzyme also accepted keto acids such as oxaloacetate and, to a lesser extent glyoxylate. Subsequent cloning of the gene revealed that it encoded a polypeptide of 359 amino acids.^45^

**Figure 6:**
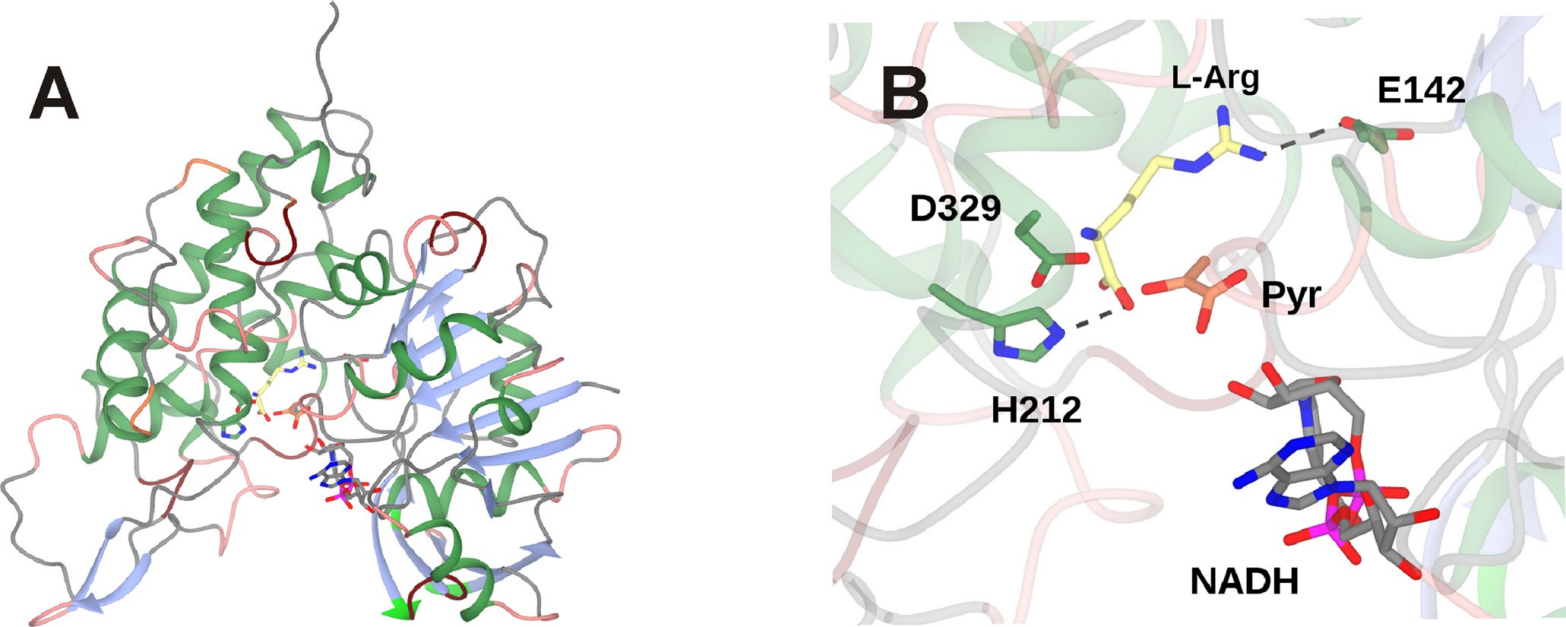
Structure of octopine dehydrogenase, an OpDH from *Pecten maximus*.^47^ **A**: Two‐domain structure of OpDH monomer showing NADH, l‐arginine (3C7C) and pyruvate (3C7D) in active site cleft between domains. **B**: Detail of OpDH active site showing interactions between l‐arginine and active site side‐chains. The positioning of the pyruvate ligand was achieved through superimposition of 3C7C and 3C7D structures.

The structure of the enzyme was determined by Asano, Rice and co‐workers,^46^ and, in common with some other AADHs, revealed a two‐domain structure with a cleft at the interface in which the nicotinamide cofactor was observed. Further information has been obtained from a structure of the related OpDH, octopine dehydrogenase, from the great scallop *Pecten maximus* (Figure 6A), as separate complexes have been obtained with the amino acids arginine and also pyruvate.^47^ These complexes have been used, along with NMR^48^ and ITC measurements,^49^ to begin to describe a mechanism for OpDH activity.

In the l‐arginine complex, among other interactions, the alpha‐carboxylate is bound to H212; the guanidinium group to the side‐chain of E142 (Figure 6B).^47^ As these residues stem from the different domains it is thought that l‐arginine binding stimulates a relative closure of the domains to prime the enzyme for the coupling reaction. In the pyruvate complex, the carboxylate of pyruvate binds to the side‐chain of Q118 and H212 makes an H‐bond with the carbonyl group. The carbonyl group is distant from the NADH cofactor, meaning that reduction of pyruvate to lactate is not observed. Mutation of Q118 to alanine gave a variant of markedly reduced activity. A sequential binding order for these two substrates was proposed based on these observations. A superimposition of l‐arginine and pyruvate complexes reveals little difference in the orientation of Q118 and H212 and permits observation of the two ligands in their binding modes (Figure 6B). However, each ligand is somewhat distant from the nicotinamide ring of the NADPH cofactor, suggesting that the active conformation, in which the imine bond between the substrate partners is reduced by hydride from the C4 atom of that ring, must incorporate different ligand binding modes.

Further evidence for the sequential, ordered binding of l‐arginine and pyruvate came from NMR studies.^48^ By monitoring five randomly selected peaks in ^15^N‐^1^H‐TROSY spectra of ^15^N labelled apo‐protein, it was observed that the binding of NADH resulted in perturbations to these chemical shifts. Further perturbations were observed upon the addition of l‐arginine to a pre‐formed OpDH‐NADH complex, but not when pyruvate was added to the same. This was strongly suggestive of l‐arginine as the second substrate, after NADH, to bind in the OpDH coupling reaction. Additional support for the mechanism was provided by isothermal calorimetry studies,^49^ which again showed measurable enthalpy changes upon the addition of l‐arginine to the OpDH‐NADH complex, but no binding when the same complex was presented with pyruvate. Interestingly, the order of “amine” and “ketone” partner binding in this reductive amination is therefore reversed from that which is observed in LeuDH and PheDH. However, although each of these experiments on OpDH sheds light on the binding order of substrates in the OpDH mechanism, no description of the chemical mechanism and the role of individual amino acid side‐chains has been reported at this stage.

### 3.2 Application of OpDH

Following the discovery of the OpDH from *Arthrobacter* sp. strain 1C, this was applied to the preparative formation of a secondary amine carboxylic acid by Asano and co‐workers.^50^ In addition to its native substrates, l‐phenyalanine and pyruvate, short chain neutral amino acids including (*S*)‐enantiomers of 2‐aminobutyric acid, norvaline, norleucine and phenylglycine were accepted as the amino acid partner substrate, with yields of between 96 and >99%, although (*S*)‐methionine was preferred. Interestingly (*S*)‐phenylalaninol, with no carboxylate group, was also converted quantitatively by the enzyme, an early indication of the promiscuous activity of the enzyme for amine substrates. Glyoxylic acid and 2‐ketobutyric acid were accepted in addition to pyruvic acid as keto acceptors.

### 3.3 Directed Evolution of OpDHs for the Reductive Amination of Ketones

**Scheme 10:**
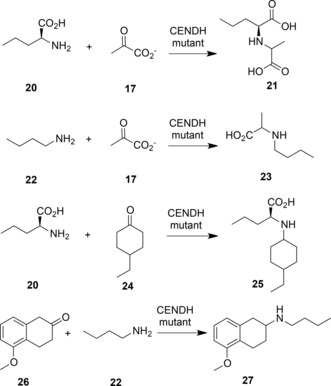
Selected reductive aminations catalysed by CENDH variants created using directed evolution.^51^ Detailed information on the enantiopurity of relevant products was not available.

**Figure 7:**
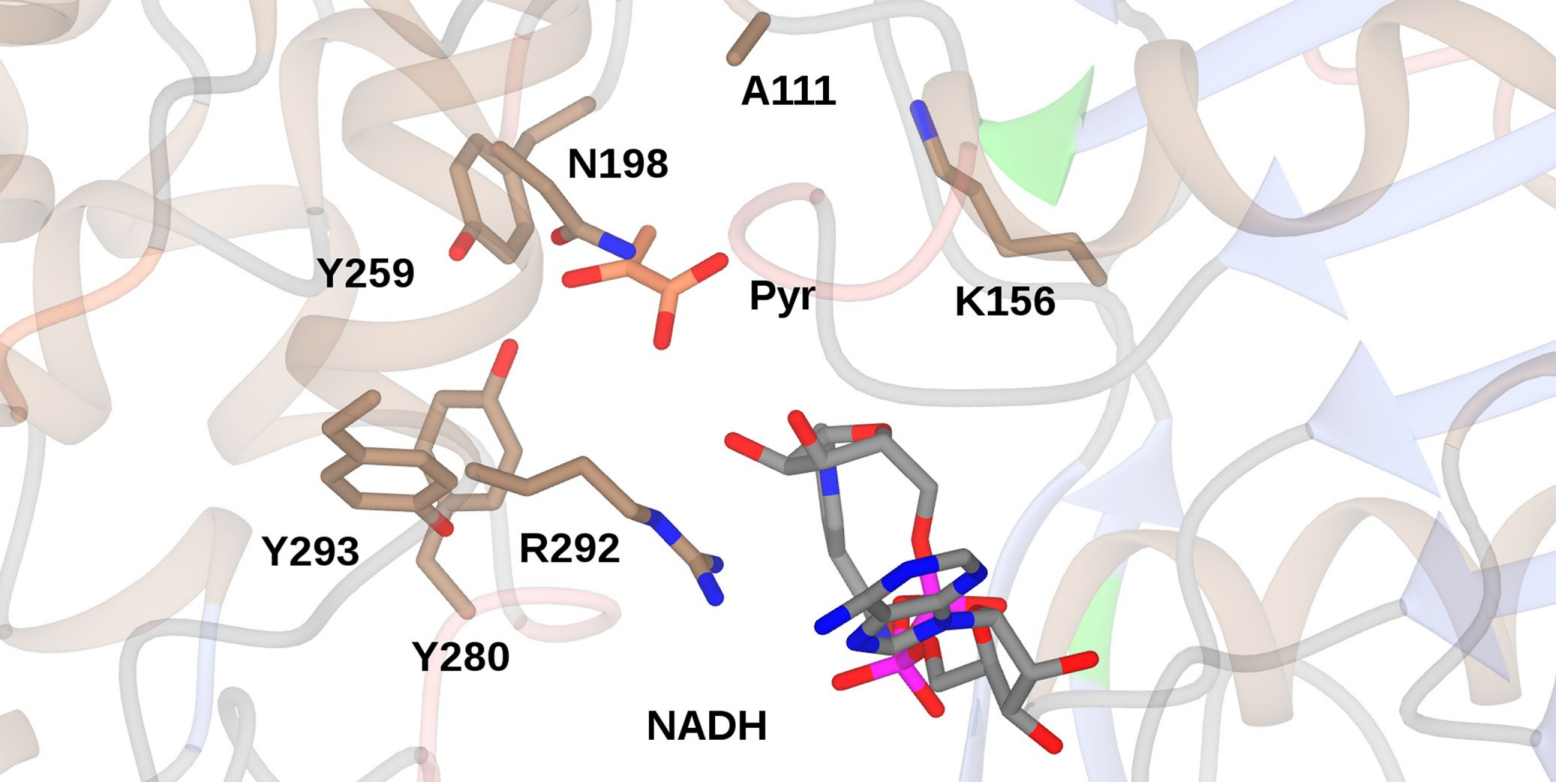
Active site of CENDH (1BG6),^46^ showing some of the active site residues targeted for mutation in directed evolution libraries leading to variants competent for the reductive amination of ketones.^51^ The position of NADH and pyruvate has been fixed using the structure of OpDH from *Pecten maximus* (3C7D).^47^.

The ability of OpDH and related enzymes to couple keto acids with amine partners larger than ammonia stimulated efforts on the part of Codexis to engineer these enzymes for the coupling of non‐carboxylated ketones and amines.^51^ Using the CENDH from *Arthrobacter* sp. strain 1C as a starting platform, an extensive programme of directed evolution experiments resulted in the first instance in CENDH variants that were not only capable of coupling pyruvate **17** to l‐norvaline **20** to give (2*S*)‐2‐[1‐(carboxyethyl)amino]pentanoic acid **21**, but also to the non‐carboxylated 1‐butylamine **22** to give 2‐(butylamino)propanoic acid **23** (Scheme 10). Further variants catalysed the reductive amination of cyclohexanone, 2‐pentanone and 2‐tetralone derivatives to a variety of amine partners to give secondary amine products (Scheme 10) with enantiomeric excesses, where relevant, of up to or greater than 99.5% *ee* One variant, which was ranked first for the coupling of cyclohexanone and butylamine, featured seven amino acid mutations (A111M/K156T/N198H/Y259M/Y280L/R292V/Y293H). An examination of these mutations in the context of the CENDH structure, reveals that the mutation sites were all within the active site cleft (Figure 7).

From these studies it is clear that the OpDH structure may be engineered for the catalysis of reductive amination reactions with ketone and amine partners, but more information on mechanism, and also the interaction of active site residues with the substrates, would assist in informing further engineering.

## 4 Imine Reductases (IREDs) and their Application in Reductive Amination Reactions

**Scheme 11:**
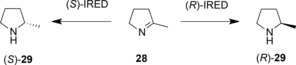
Enantiocomplementary reductions of 2‐methyl‐1‐pyrroline by (*S*)‐ and (*R*)‐IREDs from *Streptomyces* sp. GF3546 and GF5387, respectively.^52^,^53^.

Imine reductases (IREDs)^13^,^14^,^15^ were first reported to catalyse the NADPH‐dependent asymmetric reduction of cyclic imines to form chiral amine products by Mitsukura and co‐workers.^52^,^53^ Two enzymes, from different strains of *Streptomyces*, catalysed the reduction of the model imine 2‐methyl‐1‐pyrroline **28** (Scheme 11), with complementary enantioselectivity. In the last five years many other IREDs have been isolated and applied to the asymmetric reduction of a range of prochiral cyclic imines.

### 4.1 Structure of IREDs

**Figure 8:**
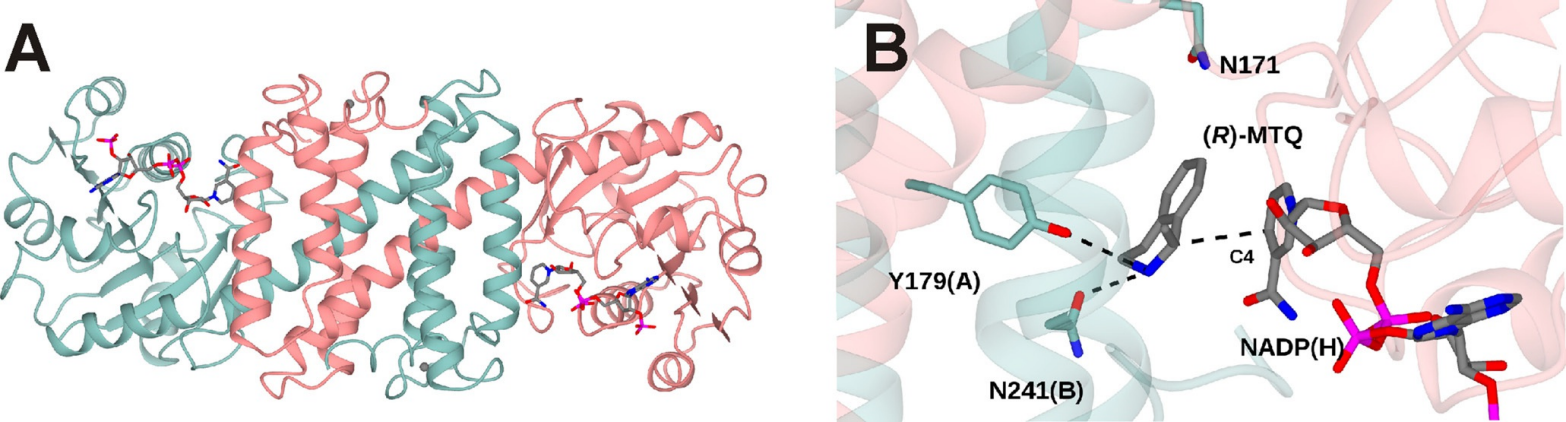
Structure of an imine reductase (IRED). **A**: IRED from *Amycolatopsis orientalis* (*Ao*IRED), showing dimeric structure of active site with NADP(H) bound at the dimer interface. **B**: Detail of active site of *Ao*IRED showing relationship between the NADP(H) cofactor and an amine product ligand (*R*)‐MTQ.^59^.

IREDs have been shown^54^,^55^,^56^ to possess a dimeric structure in which one monomer of approximately 30 kDa is intimately associated with its partner to form an active site through domain sharing. The active site is found at the interface of the N‐terminal Rossman fold of one monomer, and the C‐terminal bundle of its neighbour (Figure 8A). The two domains of each monomer are connected by a long inter‐domain helix, and the dimer structure is thought to be flexible throughout the reaction coordinate, with significant closure observed between domains upon substrate binding. The structures of IREDs are most similar to those of hydroxyisobutyrate dehydrogenases (HIBDHs), typified by PDB code 2CVZ,^57^ although domain sharing is not observed in the latter enzymes. In HIBDHs, a catalytic lysine in the active site is thought to protonate the nascent alcohol in the reductive direction.^57^ When the structures of HIBDHs and IREDs are superimposed, the lysine of 2CVZ is observed to overlap with different residues according to the IRED studied, being in some cases aspartic acid or tyrosine. A role for these residues in protonation of amine products in IRED‐catalysed reactions has been suggested,^54^,^58^ and indeed their mutation to alanine leads to enzymes of poor activity, although their role in mechanism has yet to be confirmed by structural studies. A recent structure of the IRED from *Amycolatopsis orientalis* is the first in which the amine product (*R*)‐1‐methyl‐tetrahydroisoquinoline (*R*‐MTQ) has been observed in the presence of the cofactor (Figure 8B).^59^

In this complex, the active site is indeed more closed than has previously been observed in other IREDs structures determined in the absence of a ligand, and the amine makes bonding interactions with residue side‐chains in the active site including Y179 and N241. This complex is at least suggestive of a role for these residues in ligand anchoring, and places the electrophilic carbon of the amine within a suitable distance of the C‐4 of the nicotinamide ring of NADPH for hydride exchange. The ligand is somewhat distant from the pendant asparagine residue N171 that is the structural equivalent of the catalytic lysine in HIBDH however, so the significance of the residue in this position, at least for the reduction of pre‐formed imines by IREDs, remains unclear.

### 4.2 Reductive Amination Reactions using IREDs

**Scheme 12:**
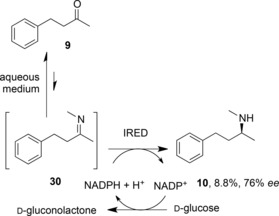
Reductive amination of 4‐phenylbutan‐2‐one by (*S*)‐IRED from *Streptomyces* sp. GF3546.^55^.

The potential of IREDs for reductive amination reactions was first described by Müller and co‐workers,^55^ who applied the (*S*)‐selective IRED from *Streptomyces* sp. GF3546 to the asymmetric reductive amination of 4‐phenyl‐2‐butanone **9** (Scheme 12), using methylammonium buffer as a source of methylamine. Although the amine equivalents were very high, and a large amount of enzyme had to be employed, a chiral amine product **10** resulted, albeit with a low conversion and modest *ee* of 8.8% and 76%, respectively. It was not clear however if the IRED catalysed a true reductive amination, both bringing the ketone and amine together in the active site, followed by NADPH‐mediated reduction, or merely reduced a pre‐formed imine **30** that had formed owing to the large excess of amine in the reaction medium.

Nonetheless, the principle of IREDs being applied to reductive amination processes had been established. Hauer and co‐workers followed this initial report with a study of bimolecular reductive amination reactions in which an IRED from *Streptosporangium roseum* was challenged with benzaldehyde in the presence of ammonia, methylamine or aniline.^60^ While reaction rates were slow with one equivalent of the amine, an increase to 10 or 50 equivalents gave, for example, a 73% conversion of benzylaldehyde and methylamine to *N*‐methylbenzylamine after 8 h in the latter case. Acetophenone and cyclohexylacetone were coupled with methylamine to give amine products with 39% and 53% conversion and 87% and 78% *ee*, respectively. In each case a 50‐fold excess of amine was required, and improved conversions were obtained at pH 9.0, favouring imine formation. NMR studies suggested that the IRED was very effective at drawing the imine intermediate from solution, as no imine intermediate in the conversion of acetophenone was detected.

**Scheme 13:**
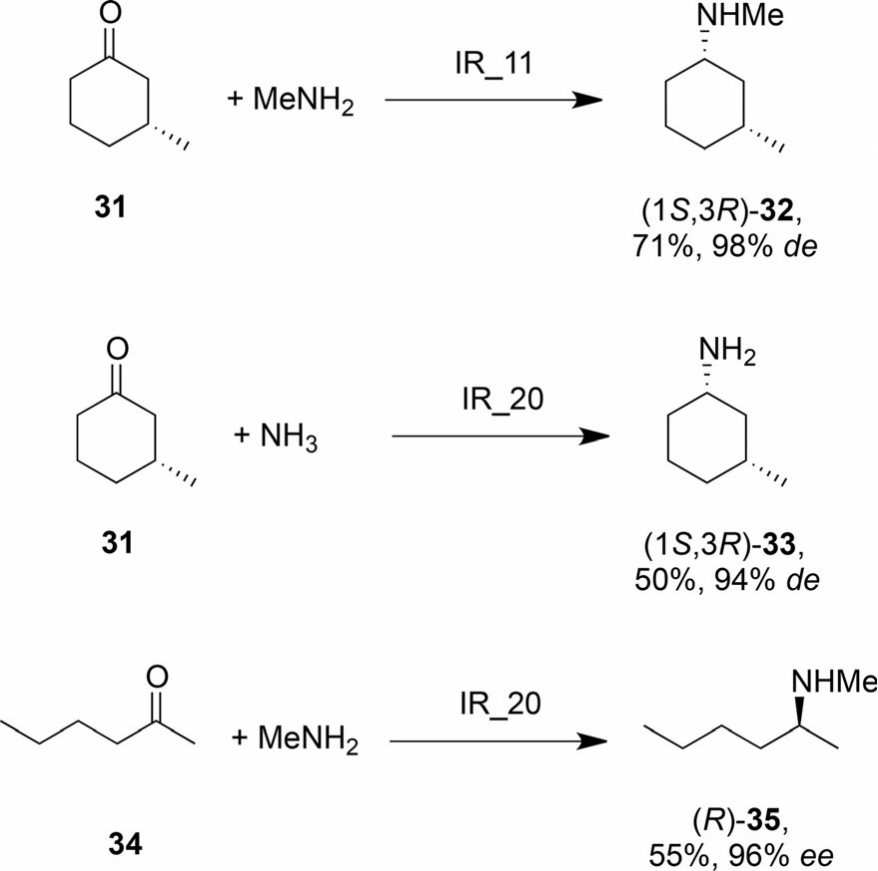
Selected reductive aminations of ketones by some of a library of IREDs discovered by Roche.^62^.

The theme of IRED‐catalysed asymmetric reductive amination was continued by the group at Roche, who had initially described a large family of IREDs, with complementary selectivity, competent for the asymmetric reduction of cyclic imines.^61^ This library of enzymes, with nine further additions, was then screened for an influence on the reductive amination of a library of ketone and amine partners including acetophenone, 2‐hexanone and cyclohexanone, with amine nucleophiles ammonia, methylamine or butylamine.^62^ Conversions were observed to vary from 10% to >90%, depending on the substrates, with aminations of acetophenone determined to be low‐yielding. Two of the superior IREDs, ‘IR_11’ and ‘IR_20’ were applied to 100 mg scale reactions. IR_11 converted ketone **31** and methylamine to **32** in 71% yield and with 98% *de* (Scheme 13); IR_20 catalysed the conversion of **31** and ammonia to **33** in 50% yield and with 94% *de* In addition, IR_20 catalysed the reductive amination of **34** with methylamine to form **35** with 55% yield and 96% *ee* In each case the amine was supplied in 12.5 molar equivalents and the reactions were again carried out at a pH of 9.3, to favour imine formation. Low activities were again attributed to the low instance of imine formation in aqueous solution, and again, little or none of the imine intermediates was detectable using NMR.

**Scheme 14:**
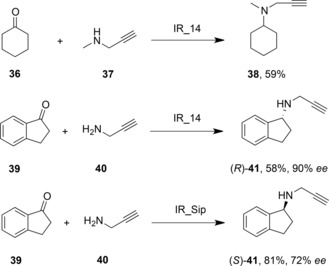
Preparation of (*R*)‐and (*S*)‐rasagiline using IREDs in reductive amination reactions.^63^.

Höhne and co‐workers further developed this work with a focus on the synthesis of pharmaceutical targets.^63^ The Roche library of IREDs was again screened for reductive amination activity, and, in addition to activities previously determined, a secondary amine **37** was employed as a nucleophile in the successful amination of cyclohexanone **36** (Scheme 14). The anti‐Parkinson's disease compound (*R*)‐rasagiline **41** was also prepared from indanone **39** and propargylamine **40** using ‘IR_14’ from the Roche library, with 58% yield and 90% *ee* In addition, another IRED, ‘IR‐Sip’ was used in the production of the (*S*)‐enantiomer of rasagiline in 81% yield and 72% *ee* In each case, the reaction was buffered at pH 9.0 and a 40‐fold excess of amine was required, again suggestive of the pre‐formation of the imine substrate as a prerequisite for the activity of the IRED.

The application of IREDs in reductive amination reactions has thus far been shown to be useful for directing the asymmetric reduction of pre‐formed imines at high pH, but any role of the enzyme in the catalysis of formation of the imine *via* a general mechanism such as that shown in Figure 1 is far from clear. However, the increasing availability of IRED sequences and structures, and the availability of high‐throughput methods of enzyme evolution, suggest that the investigation of improved IREDs for reductive amination reactions will be the subject of considerable research in the future.

## 5 Conclusions

‘Reductive aminases’ would be a considerable and potent addition to the current portfolio of enzymes available for the preparation of optically active amines. Recent research suggests that there may be numerous routes to the discovery of such activities, from the engineering of extant amino acid dehydrogenases, including opine dehydrogenases, through to the discovery of natural amine dehydrogenases, and the recruitment of imine reductases. In some cases, the mechanistic basis for the reductive amination reaction is clear; in others the role of the enzyme in the reductive amination process must be further investigated. These studies will help to inform the further engineering of reductive aminase activity for broadened substrate scope, and also for process suitability, so that the enzymes might be applied in the industrial synthesis of chiral amines.

## Biographical Information

*Mahima Sharma* is a postdoctoral research associate based in the group of Prof. Gideon Grogan at the York Structural Biology Laboratory, University of York since May 2015. She completed her DPhil in chemical biology from the University of Oxford in 2015, working under the supervision of Prof. Benjamin G. Davis at the Department of Chemistry, where she investigated the design of artificial metalloenzymes for C–C cross‐coupling reactions. Her research interests include structure determination and engineering of enzymes for biocatalytic purposes. Her current project is focussed upon discovering enzymes enabling chiral amine synthesis, in particular imine reductases (IREDs), and undertaking structural and biochemical studies thereof.

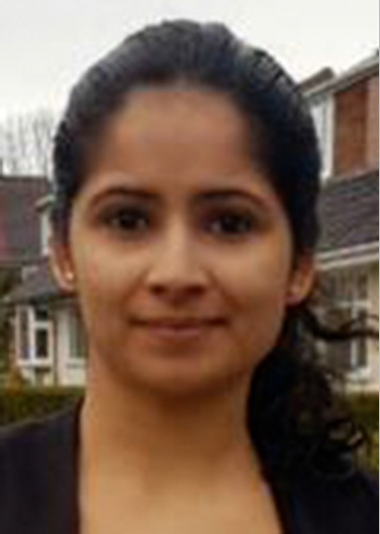

## Biographical Information

*Juan Mangas‐Sanchez* obtained his Ph.D. at the University of Oviedo (Spain) under the supervision of Vicente Gotor‐Fernandez working on new biocatalytic routes to synthesise optically active alcohols employing alcohol dehydrogenases and lipases. Then he moved to Lund University in Sweden for two years to work in Prof. Patrick Adlercreutz's group on the optimisation of chemoenzymatic processes to obtain biodiesel, tailored triglycerides and prebiotics using hydrolases. For the last two years, he has been working as a research associate at the Manchester Institute of Biotechnology in the group of Prof. Nicholas Turner. His research interests focus on the discovery, engineering, characterisation and applications of novel biocatalysts for the production of amines.

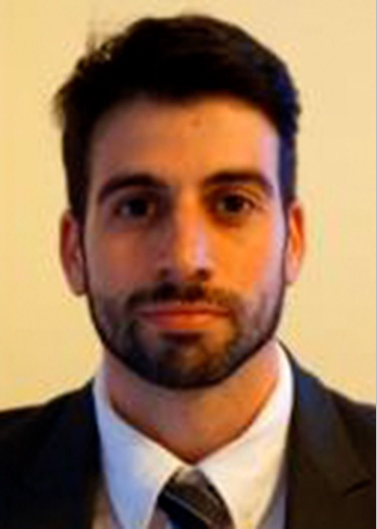

## Biographical Information

*Nick Turner* obtained his DPhil in 1985 with Professor Sir Jack Baldwin and from 1985–1987 was a Royal Society Junior Research Fellow, spending time at Harvard University with Professor George Whitesides. He was appointed lecturer in 1987 at Exeter University and moved to Edinburgh in 1995, initially as a reader and subsequently professor in 1998. In October 2004 he joined Manchester University as professor of chemical biology in the Manchester Institute of Biotechnology Biocentre (MIB: www.mib.ac.uk). He is director of the Centre of Excellence in Biocatalysis (CoEBio3) (www.coebio3.org) and a co‐director of SYNBIOCHEM, the BBSRC Synthetic Biology Research Centre. He is also a co‐founder of Ingenza (www.ingenza.com). His research interests are in the area of biocatalysis with particular emphasis on the discovery and development of novel enzyme catalysed reactions for applications in organic synthesis. His group is also interested in the application of directed evolution technologies for the development of biocatalysts with tailored functions.

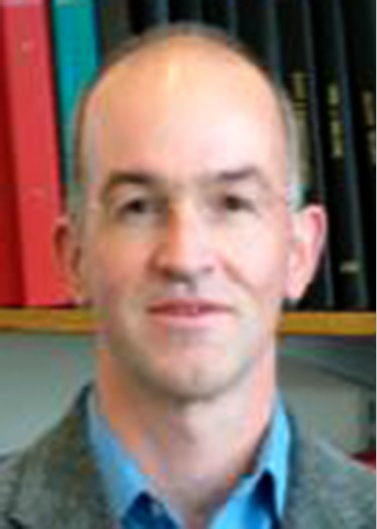

## Biographical Information

*Gideon Grogan* is professor of biochemistry at York University. He completed his Ph.D. at Exeter University with Andrew Willetts and Stanley Roberts in 1995, and a postdoc with Profs. Nick Turner and Sabine Flitsch at the University of Edinburgh between 1996 and 2000. He joined the University of York as a lecturer in 2000. His major research interests are the discovery, application and structure‐based engineering of enzymes that have potential as industrial biocatalysts. He is the author of over one hundred publications in the area of applied biocatalysis and a book *Practical Biotransformations*, a guide to enzyme technology for organic chemists.